# Efficient Differentiation of Human Induced Pluripotent Stem Cell (hiPSC)-Derived Mesenchymal Progenitors Into Adipocytes and Osteoblasts

**DOI:** 10.21769/BioProtoc.4885

**Published:** 2023-11-20

**Authors:** Martha Elena Diaz-Hernandez, Nazir M. Khan, Hicham Drissi

**Affiliations:** 1 Department of Orthopaedics, Emory University, Atlanta, USA; 2 VA Medical Center, Atlanta, USA

**Keywords:** Osteogenesis, Adipogenesis, hiPSC, Cell derivation, Mesenchymal progenitors, Trilineage differentiation, Regenerative medicine

## Abstract

Human induced pluripotent stem cells (hiPSCs) hold immense promise in regenerative medicine as they can differentiate into various cell lineages, including adipocytes, osteoblasts, and chondrocytes. Precisely guiding hiPSC-derived mesenchymal progenitor cells (iMSCs) towards specific differentiation pathways is crucial for harnessing their therapeutic potential in tissue engineering, disease modeling, and regenerative therapies. To achieve this, we present a comprehensive and reproducible protocol for effectively differentiating iMSCs into adipocytes and osteoblasts. The differentiation process entails culturing iMSCs in tailored media supplemented with specific growth factors, which act as cues to initiate adipogenic or osteogenic commitment. Our protocol provides step-by-step guidelines for achieving adipocyte and osteoblast differentiation, ensuring the generation of mature and functional cells. To validate the success of differentiation, key assessment criteria are employed. For adipogenesis, the presence of characteristic lipid droplets within the iMSC-derived cells is considered indicative of successful differentiation. Meanwhile, Alizarin Red staining serves as a marker for the osteogenic differentiation, confirming the formation of mineralized nodules. Importantly, the described method stands out due to its simplicity, eliminating the need for specialized equipment, expensive materials, or complex reagents. Its ease of implementation offers an attractive advantage for researchers seeking robust and cost-effective approaches to derive adipocytes and osteoblasts from iMSCs. Overall, this protocol establishes a foundation for exploring the therapeutic potential of hiPSC-derived cells and advancing the field of regenerative medicine.

Key features

• iMSC derivation in this protocol uses embryonic body formation technique.

• Adipogenesis and osteogenesis protocols were optimized for human iPSC-derived iMSCs.

• Derivation of iMSC from hiPSC was developed in a feeder-free culture condition.

• This protocol does not include human iPSC reprogramming strategies.

**Graphical overview**


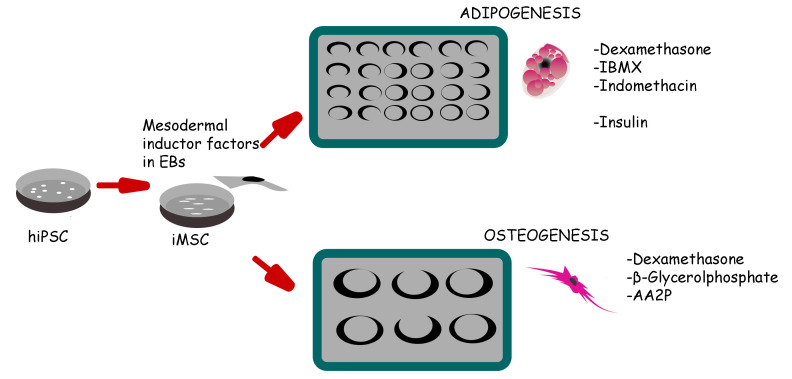


**Schematic representation of induced pluripotent stem cell (iPSC) differentiation into adipocytes and osteoblasts via mesenchymal progenitors as intermediates**

## Background

Mesenchymal stromal/stem cells (MSCs) hold immense promise in regenerative medicine as they possess the capability to differentiate into mature specialized cells. Their therapeutic utility is underscored by their replenishment property in adult tissues, although inherent variations in MSC *potency* due to donor age, tissue selection, and isolation methods necessitate further improvement for its efficient use as a therapeutic treatment. The International Society for Cell Therapy has proposed minimal criteria to identify MSCs, including adherence to plastic under standard culture conditions and the expression of specific surface markers such as CD105^+^, CD73^+^, CD90^+^, CD34^-^, CD14^-^, CD11b^-^, CD79^-^, CD19^-^, or HLA-DR (Dominici et al., 2006; Uder et al., 2018). Recently, adult MSCs have gained attention for their immunoregulatory properties, intrinsic regenerative capacity, and multilineage potential (Hoang et al., 2022; Kim et al., 2022). Although MSC derivation poses an intrinsic therapeutic utility, heterogeneity derived from donor to donor, tissue isolation, collection, and expansion methods need to be improved for their efficient use as a therapeutic treatment (Hwang et al., 2009).

Interestingly, the derivation of MSCs from human induced pluripotent stem cells (hiPSCs) offers a viable alternative (iPS-MSCs or iMSCs), presenting an avenue to study activation pathways and differentiation routes during cell commitment and terminal differentiation. Differentiation methodologies for adipocytes, cartilage, and bone have been developed by recapitulating molecular cues present in the embryo, given MSCs’ origin from the mesodermal layer (Hoang et al., 2022; Humphreys et al., 2022). Selective induction of mesodermal cells and specific iMSC subpopulations expressing distinct surface markers has been reported using culture conditions. It has been reported that iMSC subpopulations expressing CD29^+^, CD44^+^, CD73^+^, CD90, and CD105^+^ CD11b^-^, CD14^-^, CD31^-^, CD45^-^, and HLA-DR- markers have a faster proliferative capability and restrictive adipogenic ability compared to BM-MSC (Kang et al., 2015).

Here, we present detailed step-by-step protocols for the differentiation of osteoblasts and adipocytes from human iPSCs, starting from a naive pluripotent state and via the derivation of a scalable, mesenchymal-like progenitor intermediate, as described in our previous work (Khan et al., 2023). The protocols include the derivation of iMSCs from hiPSCs through embryoid bodies (EBs) formation, followed by osteoblast and adipocyte differentiation. Various assessment methods are also described to validate the maturation of functional cells. The standardized protocols provided in this study offer valuable insights into the differentiation potential of hiPSC-derived iMSCs, contributing to a deeper understanding of their therapeutic applications. By elucidating the intricacies of cell commitment and maturation processes, these methodologies may lead to further improvements in the development of regenerative therapies and disease modeling.

## Materials and reagents

**Biological materials**

1. HDFa-YK27-hiPSC human dermal fibroblast line (University of Connecticut cell and Genome Engineering Core)
2. YK27-iPSC–derived iMSCs (Emory Musculoskeletal Center, Emory University)

**Reagents**

1. mTeSR-1 media (Stemcell Technologies, catalog number: 85871)
2. Accutase in DPBS without Ca^++^ or Mg^++^ (Innovative Cell Technologies, catalog number: AT-104)
3. Dulbecco’s Modified Eagle medium (DMEM) (1×) (Gibco, catalog number: 11965-092)
4. HyClone fetal bovine serum (FBS) defined, heat-inactivated (Cytiva, catalog number: SH30070.03HI)
5. Bovine serum albumin (BSA) solution 7.5% in DPBS (Sigma-Aldrich, catalog number: A8412-100mL)
6. Penicillin streptomycin (Pen/Strep) (Gibco, catalog number: 15070-063)
7. Non-essential amino acid solution (MEM NEAA) (100×) (Gibco, catalog number: 11140-050)
8. Gelatin 0.1% in water (Stemcell Technologies, catalog number: 07903)
9. Dulbecco’s Ca^2+^ Mg^2+^ free phosphate buffered saline (DPBS) (10×) (Gibco, catalog number: 14080055)
10. Dimethyl sulfoxide (DMSO) (Sigma-Aldrich, catalog number: D8418)
11. StemLine II (Sigma-Aldrich, catalog number: SLCM2573)
12. Dulbecco’s Ca^2+^ Mg^2+^ free phosphate buffered saline (DPBS) (1×) (Gibco, catalog number: 14040117)
13. Trypsin-EDTA 0.25% (1×) phenol red (Gibco, catalog number: 25200056)
14. Trypan Blue solution (0.4%) (Gibco, catalog number: 152500-061)
15. GlutaMAX 200 mM supplement (Gibco, catalog number: 35050061)
16. Collagen I, rat tail (Gibco, catalog number: 1048301)
17. 2-propanol or Isopropanol (Sigma-Aldrich, catalog number: 67-63-0)
18. IBMX (3-isobuty-l-methyl-xanthine) (Sigma-Aldrich, catalog number: 28822-58-4)
19. Alkaline phosphatase (ALP) (Sigma-Aldrich, catalog number: 85L2-1kt)
20. 10× phosphate buffered saline (PBS) (Gibco, catalog number: 14200-075)
21. bFGF (146 a.a.) (Peprotech, catalog number: 100-18C)
22. Recombinant human BMP4 (Peprotech, catalog number: 120-05ET)
23. Dexamethasone (Stem Cell Technologies, catalog number: 72092)
24. Indomethacin (Stem Cell Technologies, catalog number: 73942)
25. β glycerol phosphate (Sigma-Aldrich, catalog number: 13408-09-8)
26. Recombinant human insulin solution (Santa Cruz Biotechnology, catalog number: 11061-68-0)
27. Oil red stock solution (Sigma-Aldrich, catalog number: O0625)
28. Recombinant human BMP4 (Peprotech, catalog number: 120-05)
29. Recombinant Human VEGF (Peprotech, catalog number: 100-20)
30. Sodium hydroxide (NaOH) (Sigma, catalog number: 484024)
31. Ascorbic-acid-2-phosphate (AA2P) (Sigma, catalog number: A8960)

**Solutions**

1. 10 μg/mL of bFGF (see Recipes)
2. 25 μg/mL recombinant human BMP4 (see Recipes)
3. 10 mM dexamethasone solution (see Recipes)
4. 10 mM indomethacin (see Recipes)
5. 20 mM IBMX (see Recipes)
6. 1 N NaOH (see Recipes)
7. 3% Oil red stock solution (see Recipes)
8. 4% paraformaldehyde (PFA) solution (see Recipes)
9. iMSC maintenance media (see Recipes)
10. 50 mg/mL ascorbic acid (AA2P) (see Recipes)
11. 1 M β glycerol phosphate (see Recipes)
12. Pre-adipocyte basal media (see Recipes)
13. iMSC expansion media (see Recipes)
14. Adipocyte media (see Recipes)
15. Insulin induction media (see Recipes)
16. Osteogenic media (see Recipes)
17. iMSC frozen media (see Recipes)
18. Collagen I rat tail coated plates (see Recipes)
19. 2% Alizarin Red staining solution (see Recipes)

**Recipes**

1. **10 μg/mL of bFGF (146 a.a.)**
  **Table:** 

Reagent	Final concentration	Quantity
5 mM HCl containing 0.1% BSA	NA	1 mL
bFGF	10 ng/μL	10 μg
  Centrifuge vial prior to opening. Avoid repeated freeze-thaw cycles. Store aliquots at -80 °C.
2. **25 μg/mL recombinant human BMP4**
  **Table:** 

Reagent	Final concentration	Quantity
5 mM HCl containing 0.1% BSA	NA	400 μL
BMP4	25 ng/μL	10 μg
  Centrifuge vial prior to opening. Avoid repeated freeze-thaw cycles. Store aliquots at -80 °C.
3. **10 mM dexamethasone solution (F.W: 392.4 g/mol) (stock solution)**
  **Table:** 

Reagent	Stock concentration	Quantity
DMSO	NA	2.54 mL
Dexamethasone	10 mM	10 mg
  As a general guide, small molecules in DMSO solution are recommended to be stored in small aliquots at -20 °C. Aliquot the solutions into working volumes to avoid freeze-thaw cycles. Protect from prolonged exposure to light.
4. **10 mM indomethacin (F.W: 357.9 g/mol) (100× stock solution)**
  **Table:** 

Reagent	Stock concentration	Quantity
DMSO	NA	2.79 mL
Indomethacin	10 mM	10 mg
  It is recommended to store DMSO aliquots at -20 °C. Aliquot the solutions into working volumes to avoid freeze-thaw cycles. Protect from prolonged exposure to light.
5. **20 mM IBMX (F.W: 222.2 g/mol) (stock 20×)**
  **Table:** 

Reagent	Stock concentration	Quantity
DMSO	NA	22.5 mL
IBMX	20 mM	100 mg
  Sterile filter with 0.2 μM pore microfilter. Aliquots are stored at -20 °C.
6. **1 N NaOH (F.W: 40 g/mol)**
  **Table:** 

Reagent	Final concentration	Quantity
H_2_O	NA	1,000 mL
NaOH	1 N	40 g
7. **3% Oil red stock solution**
  **Table:** 

Reagent	Final concentration	Quantity
Isopropanol	NA	100 mL
3% Oil red	NA	3 mg
  0.3% Oil red working solution: mix three parts of 3% oil red stock solution and two parts of distilled water and allow to sit for 10 min. Final solution is Whatman paper filtered. This solution is stable for 2 h. 3% Oil red stock solution is stable for one year.
8. **4% paraformaldehyde (PFA) solution**
  **Table:** 

Reagent	Final concentration	Quantity
1× PBS	NA	70 mL
PFA NaOH	4% 1 M	4 g 1 mL
  Dissolve PFA in a heating and stirring block (60 °C) and in a chemical hood. Adjust to 7.4 pH with 1 M HCl. Adjust to 100 mL final volume. Aliquots can be stored at -20 °C.
9. **iMSC maintenance media**
  **Table:** 

Reagent	Final concentration	Quantity
DMEM Pen/Strep MEM NEAA bFGF 100 μg/mL	NA 1× 1× 5 ng/mL	440 mL 5 mL 5 mL 25 μL
FBS defined	10%	50 mL
10. **50 mg/mL AA2P**
  **Table:** 

Reagent	Stock concentration	Quantity
Ascorbic-acid-2-phosphate (AA2P) Milli-Q water	50 mg	1 g 20 mL
  To dissolve the AA2P, prewarm the water at 37 °C.
11. **1 M β glycerol phosphate (F.W: 306.11 g/mol)**
  **Table:** 

Reagent	Stock concentration	Quantity
β-Glycerol phosphate Milli-Q water	1 M	1 g 3.26 mL
12. **Pre-adipocyte basal media**
  **Table:** 

Reagent	Final concentration	Quantity
DMEM FBS defined GlutaMAX Pen/strep	NA 5% 2 mM 1×	218 mL 2.25 mL 2.25 mL 2.25 mL
13. **iMSC expansion media**
  **Table:** 

Reagent	Final concentration	Quantity
StemLine II FBS defined Pen/strep VEGF^©^ hrBMP4^©^	NA 10% 1× 25 ng 25 ng	45 mL 5 mL 0.5 mL vary^©^ vary^©^
  ^©^Supplement the iMSC media with 25 ng/mL of hrBMP and 50 ng/mL of VEGF only to the media required for media change.
14. **Adipocyte media**
  **Table:** 

Reagent	Final concentration	Quantity
DMEM	NA	218.2 mL
FBS 10 mM Dexamethasone 20 mM IBMX Insulin solution 10 mM indomethacin	10% 1 μM 0.5 mM 10 μg/mL 100 μM	25 mL 25 μL 6.25 mL 25 μL 2.5 mL
15. **Insulin induction media**
  **Table:** 

Reagent	Final concentration	Quantity
DMEM Pen/Strep Insulin solution	NA 1× 10 μg/mL	225 mL 2.25 mL 22.5 μL
16. **Osteogenic media**
  **Table:** 

Reagent	Final concentration	Quantity
DMEM FBS defined 10 mM Dexamethasone 50 mg/mL ascorbic acid 1 M β-glycerophosphate Pen/Strep	NA 10% 0.1 μM 50 μg/mL 10 mM 1×	43.5 mL 5 mL 0.5 μL 50 μL 0.5 mL 0.5 mL
17. **iMSC frozen media**
  **Table:** 

Reagent	Final concentration	Quantity
iMSC maintenance media FBS DMSO	10% 80% 10%	1 mL 8 mL 1 mL
18. **Collagen I rat tail coated plates**
  Plates are rinsed twice with 1× PBS before use and kept at room temperature.
  **Table:** 

Reagent	Final concentration	Quantity
Collagen 1 rat tail 3 mg/mL Acetic acid 10× PBS	2 μg/cm^2 ^ 20 mM	Vary Vary
  Perform manipulation of the collagen on ice (2–8 °C) as gelling may occur rapidly at room temperature.
  Calculate the Collagen 1 rat tail volume needed:
  20 μg/mL collagen × final volume = stock collagen I rat tail (μg/mL)
  Calculate the volume of 20 mM acetic acid needed:
  Final volume needed-Volume of Collagen1 stock.
  100 μL of the solution is added to each well of the 24-well plate.
  Add the solution to the plates. Incubate at room temperature for 1 h. Do not freeze the Collagen 1 rat tail.
19. **2% Alizarin Red staining solution**
  **Table:** 

Reagent	Final concentration	Quantity
Alizarin Red Milli-Q water	40 mM	6.846 g 500 mL
  Adjust the pH to 4.2 with 1 N NaOH.

**Laboratory supplies**

1. 15 mL conical centrifuge tubes (Thermo Scientific, catalog number 339650)
2. 50 mL conical centrifuge tubes (Thermo Scientific, catalog number 339652)
3. 6 well-cell culture plate polystyrene (Costar, catalog number: 3506)
4. 96 well-cell culture plate polystyrene (Costar, catalog number: 3595)
5. 24 well-cell culture plate polystyrene (Costar, catalog number: 3524)
6. Cell culture dish sterile 60 mm ×15 mm (Greiner bio-one, catalog number: 628 160)
7. EZ Flow syringe filter pore size 0.22 μm (Foxx Life Sciences, catalog number: 378-2415-OEM)
8. Cell lifter polyethylene (Costar, catalog number: 3008)
9. 2.0 mL cryogenic vials (Corning, catalog number: 430661)
10. Whatman Grade 1 filter paper (Sigma-Aldrich, catalog number: WHA1001125)
11. BD 10 mL syringe Luer-Lok tip (BD, catalog number: 309604)
12. EZFlow^®^ 33 mm STERILE syringe filter, 0.2 µm (Stellar Scientific, catalog number: 379-2415-OEM)

## Equipment

1. Bright-line chamber and 0.4 mm cover glass (Electron Microscopy Sciences, catalog number: 63514-11)
2. Corning LX cool cell freezing system for cryogenic vials (Corning, catalog number: 432004)
3. Plate reader (SpectraMax^®^ iD3, Molecular Devices, catalog number: 735-0391)
4. Tissue culture incubator, 5% CO_2_ atmosphere (Forma Scientific, model: 3110)
5. Laminar flow hood (StreilGARD III Advance, The Baker Company, model: SG603)
6. Light microscope for live imaging (Nikon, model: TMS 215135)

## Procedure

- **MSC derivation from hiPSC using 3D embryonic body formation method**
  In this protocol, we use the formation of embryonic bodies (EBs) to facilitate the commitment of the hiPSCs (HDFa-YK27-hiPSC) to the mesodermal lineage. Once EBs have formed, iMSC enrichment is achieved by induction with mesodermal factors and later selection of specific MSC population (YK27-iPSC–derived iMSCs).
  1. To achieve proper cell density for iMSC derivation, we strongly recommend using three wells of the 6-well plate or one 6 cm dish of hiPSC culture. Wells and dish(es) must be 80% confluent.
  2. To perform EB formation, dissociation of hiPSCs colonies is achieved by Accutase-PBS diluted (1:1 ratio) treatment. Use 1 mL of this solution (Accutase) for one well of the 6-well plate, whereas 3 mL is needed for a 6 cm dish.
  3. Colonies are partially dissociated at room temperature for 3 min. After 3 min, slow down cell dissociation by adding 1 mL of mTeSR-1 media; then, lift clumps of hiPSCs cells and transfer into a 15 mL conical tube.
  4. Centrifuge tubes at 300× *g* for 5 min.
  5. Aspirate the supernatant from the tube; the pellet should be visible at the bottom. Then, add 0.5 mL of mTeSR-1 media to each well of a 6-well plate (for each 6 cm dish, add 1 mL of mTeSR-1 media).
  6. Gently resuspend the pellet. Clumps of 2–3 cells together are still expected.
  7. Perform cell counting (see the hiPSC counting method in General notes and troubleshooting).
  8. A centrifugation of 300× *g* is required to adjust cell number to 15,000/15 μL in mTeSR-1 media. We recommend having 3.0 × 10^5^ cell density as a minimum to obtain 20 EBs.
  9. Dispense 15 μL of cell suspension into a 10 cm Petri dish. The use of a 20 μL pipette tip is preferred for easy cell dispensing. iPSC drops should be at a certain distance from each other to prevent them from fusing together (Figure 1A).
    **Figure 1.**
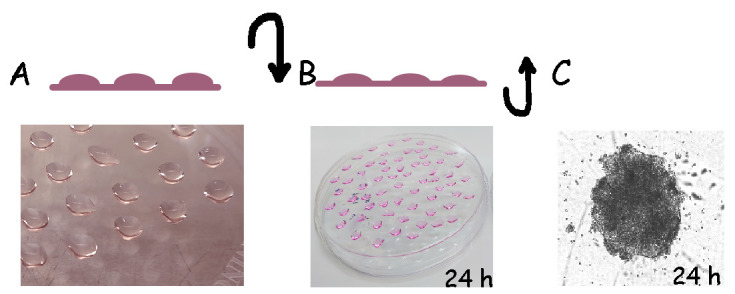
Graphical representation of step-by-step method used for embryonic body (EB) formation. A. Human induced pluripotent stem cells (hiPSCs) are dissociated and seeded in high-density drops in a 10 cm Petri dish. B. The Petri dish is inverted upside down immediately after preparing drops and incubated for 24 h at 37 °C. C. Plates are inverted back and cultured for an additional 24 h to allow the formation of EBs, which are counted manually and transferred to gelatin plates.
  10. Close the lid of the Petri dish and then flip the plate over. Keep the dish inside the incubator for 16 h at inverted position (Figure 1B).
  11. After incubation, flip the Petri dish back and carefully add 8 mL of mTeSR-1 media. Return the plate to the CO_2_ incubator for an additional 24 h at 37 °C (Figure 1C).
  12. Transfer the EBs into a 6 cm dish coated with 1% gelatin. The use of the 1 mL pipette tip is preferred for easy handling of EBs.
  13. Replace the mTeSR-1 media with iMSC expansion media (see Recipe 14) and supplement it with VEGF and BMP4 factors (see Recipe 2).
  14. EBs have adhered to the plate after 24 h. However, some EBs will still be floating. A mix of MSC precursors should appear in the next days. Maintain the media and factors for a total of three days.
  15. Replace the iMSC expansion media with iMSC maintenance media (see Recipe 10) for the next 5–7 days.
  16. Once the dish is confluent, pass the iMSCs mix population using the Accutase-PBS method, as previously described. At this point, there is no need to dissociate EBs in single-cell suspension. Transfer EB aggregates and floating cells to two dishes coated with gelatin to expand. Label dishes as *passage number 0*.
  17. iMSCs should be passaged when they reach confluency. From P1, they should be passaged by the trypsinization method (see General notes and troubleshooting). Once they reach P4, perform gene expression and osteo, adipo, and chondrogenesis and cell surface analyses to select specific cell populations, if required.
  18. iMSCs are cryo-preserved for further validation and expansion (See General notes and troubleshooting, Recipes).
- **Recovery and maintenance of human iMSCs**
  1. Transfer iMSCs from cryovials to the hood in dry ice. Cell thawing should be completed in a short time to maximize cell survival. Immerse frozen cryovials in a clean water bath at 37 °C; once the chunk of ice is melted (approximately 1 min), gently transfer the cells to a sterile 15 mL conical tube containing 9 mL of DMEM media.
  2. Centrifuge the tubes at 400× *g* for 3 min to pellet the cells.
  3. Aspirate the media and resuspend the cells in maintenance media.
  4. For cell culture maintenance, use a 10 cm culture dish without any coating.
  5. Change media every three days until iMSCs have reached 80% of confluency.
- **iMSC cryopreservation**
  1. iMSC frozen vials are made in early passages at 1 × 10^6^/mL cell density.
  2. Prepare the iMSC frozen media (see Recipe 18) in advance and keep at room temperature.
  3. Briefly, remove maintenance media from culture dishes and wash once with 1× PBS.
  4. Perform trypsinization and cell counting (see General notes and troubleshooting).
  5. Adjust cell density to 1 × 10^6^ /mL in iMSC maintenance media.
  6. Centrifuge cell suspension at 400× *g* for 5 min.
  7. Resuspend pellet in frozen media to have 1 × 10^6^/mL, immediately transfer into labeled cryovials, and place on the Corning LX cool cell freezing system. Keep at -80 °C overnight.
  8. Cryovials are ready for long-term storage at the liquid nitrogen tank.
- **Preadipocyte growth**
  1. To promote adipogenic differentiation, treatment should start once MSCs are in the arrest phase of the cell cycle (Zebisch et al., 2012). Although there is no specific recommendation to the plate size, 24-well plates are preferred for the adipocyte assay and 4,000 cells/cm^2^ is recommended as initial seeding density. Collagen I from rat tail is used to coat the plates (see Recipe 19). Keep in mind that experimental duplicates or triplicates and control wells (no-treatment group) should be included for all experiments.
  2. Growth and preadipocyte expansion are achieved by using pre-adipocyte basal media (see Recipe 13) with media changes every other day until cells reach 100% confluency.
  3. During preadipocyte growth, iMSCs do not show major changes in morphology. At this stage, iMSCs are seen as fibroblast-like cells. Maintain iMSCs, preadipocytes, and adipocytes in 5% CO_2_, 95% humidity and 37 °C.
- **Adipocyte induction**
  1. Adipocyte induction is promoted at iMSC and preadipocytes 100% confluency and in intervals/cycle between the adipocyte media (see Recipe 15) for 3–4 days, followed by insulin induction media (see Recipe 16) for an additional three days.
  2. Perform three repeated interval-cycles (adipocyte media: insulin media); pay special attention during media changes to avoid drying and detachment of cells from the plastic surface between media changes. During adipocyte induction, preadipocytes change their morphology to a spherical shape, and lipid droplets inside of the cytoplasm are seen. Multiple induction series can be performed until 95% of the cells are committed to adipogenic lineage.
  3. Changes in iMSC morphology are observed approximately at day 7, and cells become highly responsive to lipogenic and lipolytic hormones and accumulate high levels of lipogenic enzymes (Smith et al., 1988). Monitor terminal differentiation by brightfield microscopy, through changes from an elongated to a round shape and an evident presence of lipid vacuoles, which increase in number and size covering the whole well.
  4. Multiple induction treatments resulted in more than 95% of the cells committing to this lineage, and the lipid vacuoles continued to develop over time, coalesced, and eventually filled the cell. These adipocytes remained healthy in culture for at least three months.
- **Oil red staining**
  Once the terminal adipocyte maturation is achieved, stain lipid vacuoles of the adipocytes with 0.3% oil red solution.
  1. Fix adipocytes in 4% PFA for 15 min at room temperature, followed by one wash with 1× PBS.
  2. Remove the PBS, add 250 mL of 60% isopropanol, wait for 30 s, and aspirate the solution. Repeat the 60% isopropanol wash and then let the plate dry completely at room temperature (takes approximately 3 min).
  3. Add 200 μL of 0.3% oil red working solution to each well, including experimental and control groups (non-induced group).
  4. Incubate the plate at room temperature and monitor it under the microscope for red color precipitates, which can be visualized after 15 min of 0.3% of oil red staining (Figure 2).
    **Figure 2.**
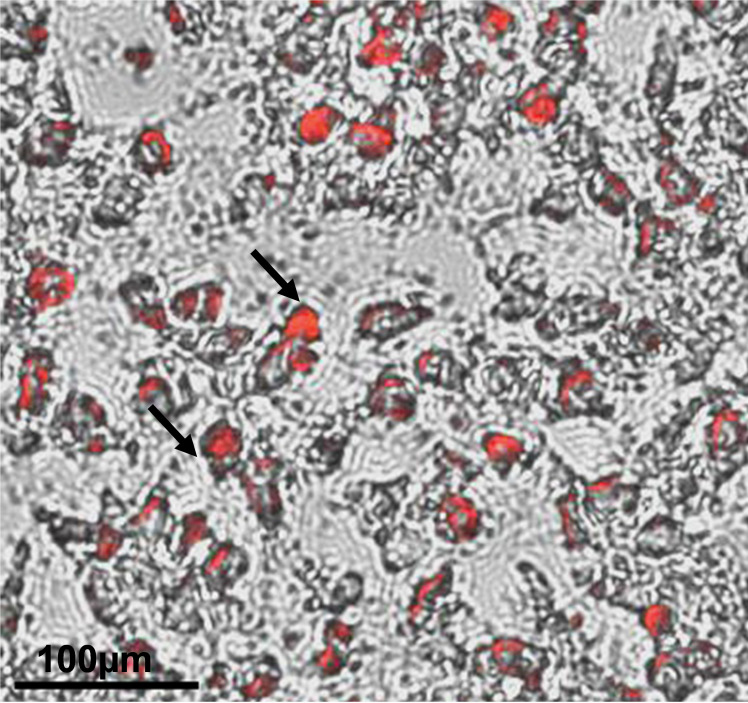
Differentiation of human induced pluripotent stem cell (hiPSC)-derived mesenchymal progenitor cells (iMSCs) into adipocytes was determined by oil red staining. Accumulation of lipid droplets as shown by the pointed arrows indicate adipocyte differentiation of iMSCs at 21 days of differentiation. Scale bar = 100 μm.
  5. Red precipitates’ area and dots (lipid vesicles) are strong in color compared to the other cell compartment areas and are an indication to stop the reaction (approximately 20 min). After staining, remove the 0.3% oil red staining solution and wash the plate once with distilled water; visualize under a microscope to be sure about proper staining. If staining is not optimal, incubate the plate with 0.3% Oil red solution for some additional time.
  6. After proper staining, remove the 0.3% oil red staining solution and wash the plate with distilled water until the water is colorless.
  7. Visualize the plate under the microscope for visible dark red color oil droplets (Figure 2).
- **Osteogenic differentiation of iMSCs**
  1. Osteogenic differentiation of iMSC can be achieved in monolayer cultures at 4,000 cells/cm^2^. 6-well plates are preferred for the osteogenic assay, but 12-well plates can also be used. From our experience, coating wells with Collagen I rat tail (see General notes and troubleshooting) decreased cell peeling during the differentiation process.
  2. After plating the iMSCs, culture the cells in MSC maintenance media (see Recipe 10) for 24 h at 37 °C, 5% CO_2_. As in adipogenesis, the treatment is preferred once MSCs are in cell cycle arrest.
  3. After 24 h of incubation, change MSC media to osteogenic media (see Recipe 17) and maintain for three days.
  4. Change induction media every 3–4 days for a total of 14–21 days.
  5. Maintain osteogenic cultures until changes in cell morphology are visualized under brightfield microscopy (after the third week of osteogenic treatment).
  6. Keep in mind to have experimental groups in duplicate or triplicates. Additionally, an undifferentiated control group (no-treatment group) should be included in the experiments.
- **Alizarin Red staining**
  Extracellular calcium deposits stain positive for Alizarin Red solution.
  1. Fix the osteogenic cultures with 4% PFA (see Recipe 9) for 15 min at room temperature.
  2. Wash the wells three time with distilled water for 5 min each.
  3. Add 700 μL of filtered 0.2% Alizarin Red staining solution to each well and incubate for 30 min at room temperature. After 30 min, monitor the staining under the microscope; cells will change from bright orange to red. If required, increase the incubation time for additional staining and periodically monitor cells until a complete red color develops (Figure 3). Keep in mind that undifferentiated control cells appear slightly reddish.
    **Figure 3.**
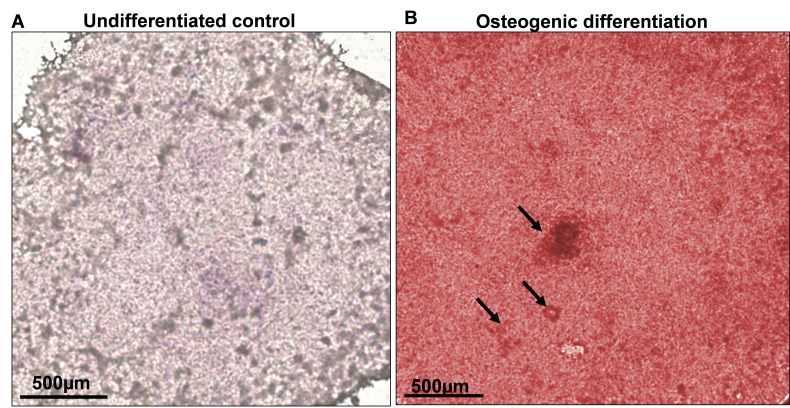
Osteoblast differentiation of human induced pluripotent stem cell (hiPSC)-derived mesenchymal progenitor cells (iMSCs) was determined by Alizarin Red S staining. Microscopic view of staining on day 21 of osteogenic differentiation. A. Undifferentiated control did not show any staining with Alizarin Red. B. Osteogenic media induced a strong red color staining, indicating the differentiation into osteoblast. The mineralization is shown by the arrow pointing towards nodule formation. Scale bar = 500 μm.
  4. After proper staining, remove the staining solution and wash the wells three times for 5 min each with distilled water.
  5. Keep the wells with distilled water for image acquisition.
- **Alkaline phosphatase staining**
  Osteoblasts form aggregates or nodules, which show increased expression of alkaline phosphatase. We followed the protocol from Sigma-Aldrich with some modifications:
  1. Culture the cells as described for osteogenesis.
  2. Prepare diazonium salt solution: dissolve one capsule of Fast violet B in 48 mL of pre-warmed water (18–26 °C).
  3. Add 2 mL of Naphthol AS-MX phosphate alkaline solution to the previous diazonium salt solution (ALP staining solution).
  4. Carefully, remove the media from the wells and fix the wells with 4% PFA (see Recipe 9).
  5. Add 1 mL of ALP staining solution to each well and incubate in the dark for 15–30 min at 37 °C.
  6. Rinse the wells two times for 5 min with tap water.
  7. Image using a light microscope.
  8. Mineralization can also be assessed by colorimetric detection at 405 nm.

## Data analysis

1. Colorimetric measurements are done in triplicates using a plate reader at 405 nm.
2. Present the data as mean ± S.E.M. of at least three independent samples. Perform statistical comparisons between untreated and growth factor–treated groups using a two-tailed Student’s *t*-test. Significance is assigned to P values < 0.05.

## General notes and troubleshooting

**General notes**

1. Determination of the cell number:
  Cell counting is performed in 100 μL aliquots of cell suspension and diluted in 100 μL of trypan blue staining solution. 10 μL of stained cells is transferred into a Neubauer chamber and cell counting is performed at the 10× objective. Counting is repeated in the second chamber, and cells average is used to calculate total number of cells per milliliter. Cell concentration is adjusted by centrifugation or dilution to obtain the desired cell density for adipogenesis or osteogenesis outcomes.
2. Determination of the hiPSC cell number:
  Cell counting is performed in a 100 μL aliquot of the hiPSCs suspension. A 100 μL aliquot of hiPSC clumps (3–4 cell aggregates) is completely dissociated by pipetting up and down to get a single-cell suspension and then diluted in 100 μL of trypan blue staining solution to a 1:1 dilution. 10 μL of the stained single cells is transferred into a Neubauer chamber, and cell counting is performed at the 10× objective. Counting is repeated in the second chamber, and cells average is used to calculate total number of cells per milliliter. Cell concentration is adjusted by centrifugation or dilution to obtain the desired cell density for EBs formation.
3. Cell trypsinization:
  Briefly, maintenance media is removed from culture dishes and washed once with 1× PBS; then, Trypsin-EDTA solution is added and incubated for 3–5 min at 37 °C. After cell detachment, trypsin is inactivated by adding maintenance media. Cells are transferred to a 50 mL conical tube. iMSCs are centrifugated at 400× *g* for 5 min and resuspended.
4. iMSC cryopreservation:
  iMSCs frozen vials are made in early passages at 1 × 10^6^ /mL cell density and passage number is documented.

**Troubleshooting**

Problem 1: EB formation. Embryonic body is disintegrated.

Possible cause: issues with cell density. A high number of cells can prevent cell aggregation. Meanwhile, a low cell number can form small EBs that will take more time to grow, which can affect the number of cells committed to the mesodermal lineage.

Solution: confirm initial cell density. Also, different cell densities can be tested to confirm the adequate cell number required for EBs formation. For iMSC derivation, we strongly recommend using three wells of the 6-well plate or one 6 cm dish for proper cell density. Wells and dish(es) must be 80% confluent.

Problem 2: Recovery and maintenance of human iMSC. Low rate of cell recovery.

Possible cause: iMSCs were harvested at the stationary phase of the cell cycle.

Solution: cells need to be collected in the growing phase to have good cell quality after thawing. Cryopreservation of iMSCs should be done at 80%–90% confluency.

Be sure that iMSCs are completely mixed with the cryopreservation media.

Dispensing of cells needs to be done gently by slowly dropping to avoid cell death excess.

Problem 3: Adipogenesis and osteogenesis peeling off in monolayer cultures.

Possible cause: excessive force during media changes and dryness of the monolayer cells.

Solution: to avoid disturbing the seeded cells, place the plate in a horizontal position during transfer from hood to CO_2_ incubator.

Aspirate the media from the edges using the manual pipette.

We also recommend leaving some media (just to protect the cells from dryness), e.g., approximately 250 μL for one well of the 6-well plate. Avoid handling the plate in a vertical position.
